# S-Adenosylmethionine Treatment of Colorectal Cancer Cell Lines Alters DNA Methylation, DNA Repair and Tumor Progression-Related Gene Expression

**DOI:** 10.3390/cells9081864

**Published:** 2020-08-09

**Authors:** Sára Zsigrai, Alexandra Kalmár, Zsófia B. Nagy, Barbara K. Barták, Gábor Valcz, Krisztina A. Szigeti, Orsolya Galamb, Titanilla Dankó, Anna Sebestyén, Gábor Barna, Vanessza Szabó, Orsolya Pipek, Anna Medgyes-Horváth, István Csabai, Zsolt Tulassay, Péter Igaz, István Takács, Béla Molnár

**Affiliations:** 1 Department of Internal Medicine and Oncology, Semmelweis University, 1083 Budapest, Hungary; kalmar.alexandra@med.semmelweis-univ.hu (A.K.); nagy.zsofia@med.semmelweis-univ.hu (Z.B.N.); molnar.barbara_kinga@med.semmelweis-univ.hu (B.K.B.); valcz.gabor@med.semmelweis-univ.hu (G.V.); szigeti.krisztina_andrea@med.semmelweis-univ.hu (K.A.S.); orsg1@yahoo.com (O.G.); igaz.peter@med.semmelweis-univ.hu (P.I.); takacs.istvan@med.semmelweis-univ.hu (I.T.); molnar.bela1@med.semmelweis-univ.hu (B.M.); 2 MTA-SE Molecular Medicine Research Group, Hungarian Academy of Sciences, 1051 Budapest, Hungary; tulassay.zsolt@med.semmelweis-univ.hu; 3 1st Department of Pathology and Experimental Cancer Research, Semmelweis University, 1085 Budapest, Hungary; tita.danko@gmail.com (T.D.); anna@korb1.sote.hu (A.S.); barna.gabor@med.semmelweis-univ.hu (G.B.); nesszy20@gmail.com (V.S.); 4 Department of Physics of Complex Systems, ELTE Eötvös Loránd University, 1117 Budapest, Hungary; pipeko@caesar.elte.hu (O.P.); mhanna@caesar.elte.hu (A.M.-H.); csabai@phys-gs.elte.hu (I.C.); 5 Department of Internal Medicine and Hematology, Semmelweis University, 1088 Budapest, Hungary

**Keywords:** colorectal cancer, S-adenosylmethionine, genomic stability, EMT, DNA methylation, epigenetics, cancer therapy

## Abstract

Global DNA hypomethylation is a characteristic feature of colorectal carcinoma (CRC). The tumor inhibitory effect of S-adenosylmethionine (SAM) methyl donor has been described in certain cancers including CRC. However, the molecular impact of SAM treatment on CRC cell lines with distinct genetic features has not been evaluated comprehensively. HT-29 and SW480 cells were treated with 0.5 and 1 mmol/L SAM for 48 h followed by cell proliferation measurements, whole-genome transcriptome and methylome analyses, DNA stability assessments and exome sequencing. SAM reduced cell number and increased senescence by causing S phase arrest, besides, multiple EMT-related genes (e.g., *TGFB1*) were downregulated in both cell lines. Alteration in the global DNA methylation level was not observed, but certain methylation changes in gene promoters were detected. SAM-induced γ-H2AX elevation could be associated with activated DNA repair pathway showing upregulated gene expression (e.g., *HUS1*). Remarkable genomic stability elevation, namely, decreased micronucleus number and comet tail length was observed only in SW480 after treatment. SAM has the potential to induce senescence, DNA repair, genome stability and to reduce CRC progression. However, the different therapeutic responses of HT-29 and SW480 to SAM emphasize the importance of the molecular characterization of CRC cases prior to methyl donor supplementation.

## 1. Introduction

Colorectal cancer (CRC) is the third most common cancer worldwide, and the second most common cause of cancer-related death according to a Globocan 2018 survey [1].

It is believed that in the case of CRC, tumor initiation and progression are the results of acquired genetic and epigenetic alterations during a series of cell divisions [2,3]. Regarding the complexity of these mechanisms, CRC is considered a heterogeneous entity [4]. Besides the application of the TNM staging system or defining major mutations (e.g., *KRAS*, *BRAF*), the classification of CRC based on molecular traits can help to predict prognosis and therapy response [1,5].

Loss of genomic stability appears to be the major driving force for tumorigenesis [3,6], that can be caused by chromosomal instability (CIN); inactivation of mismatch repair genes, known as microsatellite instability (MSI); and aberrant DNA methylation (CpG island methylator phenotype; CIMP) [7]. CRC can also be classified into four different consensus molecular subtypes (CMS) based on gene expression alterations. CMS1 is the “MSI immune” subgroup, CMS2 is the “canonical”, CMS3 is the “metabolic” and CMS4 is the “mesenchymal” class, while samples with mixed features are considered as transition phenotypes [8].

DNA methylation is one of the most intensively studied epigenetic modifications [9]. Methyl (CH3) group addition mostly occurs on cytosines, followed by a guanine (called CpG dinucleotides), that are usually concentrated in the promoter region of genes [9]. It is a dynamic and reversible biochemical process, and thereby can be a potential therapeutic target [10]. Malignant cells show major disruptions in their DNA methylation patterns [11] including genome-wide decrease in DNA methylation levels and increased methylation of promoter-specific CpGs [12]. While global DNA hypomethylation can activate the transcription of oncogenes [13] leading to the above-mentioned genomic instability, DNA methylation in promoter regions is an effective way of silencing tumor suppressors.

S-adenosylmethionine (SAM) serves as a major methyl donor molecule in these transmethylation reactions [13]. SAM is synthesized in the cytoplasm of every mammalian cell from methionine and ATP, via the methionine cycle [14]. It is an approved nutritional supplement, generally used to treat many diseases with limited documented toxicities [15]. Nevertheless, based on the results of animal and cell culture experiments, SAM has been proven to have an inhibitory effect on certain tumors including osteosarcoma [16], hepatic [17], gastric [18], prostate [19], breast [20] and colorectal cancers [21] as well.

Besides its role in DNA methylation, SAM—as a universal methyl donor molecule—can also methylate RNA, proteins and phospholipids [14,22]. Moreover, it is involved in polyamine synthesis, transsulfuration and aminopropylation, which makes it become one of the most frequently used enzyme-substrates, following ATP [14,22].

In order to explore the above-mentioned inhibitory effect of SAM treatment in CRC *in vitro*, we selected two colorectal adenocarcinoma cell lines, that were listed in different CMSs [8,23]. HT-29 cells are clustered in CMS3 [23], while SW480 cells are classified as CMS4 [8,23]. The basic methylation status of the two cell lines is also proved to be different on the basis of the CIMP 1 and 2 panels, suggested by Issa and Weisenberger et al., whereas HT-29 is CIMP+ and SW480 is CIMP- [23].

In the present study, we aimed to analyze DNA methylation alterations with NGS (next-generation sequencing)based methods or pyrosequencing and focused on genetic, transcriptomic, and consecutive functional changes with a special interest in tumor progression, genomic instability and DNA repair processes. We were also interested in whether the different molecular features between the two cell lines can influence the effect of SAM treatment.

## 2. Materials and Methods

### 2.1. Cell Cultures

HT-29 (ATCC HTB-39) and SW480 (ATCC CCL-228) human colon adenocarcinoma cell lines were cultured at 37 °C in a 5% CO_2_ humidified atmosphere and maintained in RPMI 1640 medium (Biosera, Ringmer, UK), supplemented with 10% fetal bovine serum (Biosera), 80 mg/2mL gentamycin (Sandoz GmbH, Kundl, Austria) and 2 mM l-glutamine (Biosera).

HT-29 and SW480 cells were seeded in triplicates either in 6-well plates (Sarstedt, Nümbrecht, Germany) at a density of 12.5 × 10^4^ cells/well in 2.5 mL growth medium or in 96-well plates with a density of 3 × 10^4^ cells/well in 100 μL for sulforhodamine B (SRB) cell proliferation assay. Based on the literature data [18,24,25] and our preliminary cytotoxicity tests, SAM treatment (New England BioLabs, Ipswich, MA, USA) was carried out in two different concentrations (0.5 mmol/L and 1 mmol/L) with its direct addition to the medium of HT-29 and SW480 cells, in parallel to non-treated control cultures. Cells were harvested with TrypLE Express (Thermo Fisher Scientific, Carlsbad, CA, USA) after 48 h treatment and washed with 1× phosphate-buffered saline (PBS) solution. Trypan blue dye exclusion technique was applied to monitor the cell number and viability. Finally, cells were resuspended with β-mercaptoethanol (Sigma-Aldrich, St Louis, MO, USA) containing buffer RLT (Qiagen, Hilden, Germany) in 1:100 dilution for whole genomic expression analysis or with 1× PBS.

### 2.2. Cell Proliferation Analysis with SRB Assay

HT-29 and SW480 cells were seeded in 96-well plates and treated with SAM for 48 h. After the incubation period, cells were fixed with ice-cold trichloracetic acid for 1 h at 4 °C, followed by a washing step with tap water. Cells were dyed with 50 μL 0.4% *m/v* sulphorhodamine solution for 15 min at room temperature (RT), and the unbound dye was removed by washing the plate with 1% *v/v* acetic acid. After air-drying, 150 μL of 10 mM unbuffered Tris base was pipetted to the wells and shook for 5 min. Absorbance was measured at 570 nm wavelength with a microplate reader using Transmit software (Multiskan MCC 355, Thermo Fisher Scientific). Statistical significances (set at *p* ≤ 0.05) were assessed by Kruskal–Wallis test followed by Dunn’s multiple comparisons test using Prism8 software (GraphPad, San Diego, CA, USA).

### 2.3. Cell Cycle Analysis by Flow Cytometry

Harvested HT-29 and SW480 cells were washed with 1× PBS, then fixed in 70% ice-cold ethanol overnight at −20 °C. Cell pellet was resuspended with 1× PBS followed by RNase (Thermo Fisher Scientific) treatment. After incubation at RT for 15 min, 2 μL propidium iodide (2 mg/mL) (Sigma-Aldrich) was added to the samples. The measurements were performed with FACSCalibur bench-top flow cytometer (Becton, Dickinson and Company, Franklin Lakes, NJ, USA) and analyzed by CellQuest Pro software (Becton, Dickinson and Company). Two-way ANOVA followed by a Tukey’s multiple comparisons test was applied to determine statistical significances (*p* ≤ 0.05) between the non-treated and the treated groups using Prism8 software (GraphPad).

### 2.4. Whole Genomic Expression Analysis with Microarray Technique

After harvesting, HT-29 and SW480 cell pellets were resuspended with 350 μL RLT buffer containing β-mercaptoethanol, then total RNA was purified using an RNeasy Mini Kit (Qiagen). RNA concentration was determined by Qubit 1.0 fluorometer using an RNA HS Assay Kit (Thermo Fisher Scientific). To determine the integrity of isolated RNA, an Agilent 6000 Pico Assay Kit on Agilent 2100 Bioanalyzer system was used (Agilent Technologies, Santa Clara, CA, USA). RNA samples with RNA integrity number (RIN) ≥ 8 were further used for whole transcript expression analysis. After amplification, quantification, fragmentation and terminal labelling with the use of GeneChip WT PLUS Reagent Kit (Thermo Fisher Scientific), target hybridization to the HTA 2.0 microarray chip (Human Transcriptome Array 2.0, Affymetrix, Santa Clara, CA, USA), then washing, staining and scanning was performed as previously described by Kalmar et al. [26].

Data were processed for background subtraction, normalization and signal summarization applying the SST–RMA (signal space transformation–robust multi-array average) algorithm. TAC 4.0 (Transcriptome Analysis Console 4.0, Affymetrix) software and its built-in pathway generator were used for the evaluation of gene expression alterations. Heatmaps were made from the transcripts that showed significant (*p* ≤ 0.05) and ≥|1.4| fold change (FC) alterations in the comparison of 0 and 1 mmol/L SAM treatment. Average linkage was used for clustering, and the Euclidean method for distance measurement. The dataset of the present study was uploaded to the Gene Expression Omnibus (GEO) data repository: GEO ID: GSE152870 (https://www.ncbi.nlm.nih.gov/geo/query/acc.cgi?acc=GSE152870).

### 2.5. DNA Isolation from the Treated Cells

DNA isolation was performed with the High Pure PCR Template Preparation Kit (Roche, Mannheim, Germany) with one hour of RNAse A/T1 (Thermo Fisher Scientific) digestion. The extracted DNA concentration was measured with a Qubit dsDNA HS Assay Kit (Thermo Fisher Scientific) on a Qubit 1.0 fluorometer (Thermo Fisher Scientific) and stored at −20 °C for later analysis.

### 2.6. Global DNA Methylation Analysis with LINE-1 Pyrosequencing

Isolated DNA (~50 ng) was bisulfite converted using an EZ DNA Methylation-Direct Kit (Zymo Research, Orange, FL, USA). Long interspersed nuclear element 1 (LINE-1) retrotransposons were amplified with a PyroMark PCR Kit (Qiagen) and the specificity of the PCR product was visualized with gel electrophoresis using 2% agarose gel. Samples were prepared for pyrosequencing analysis according to the PyroMark Q24 CpG LINE-1 Handbook (Qiagen) on a PyroMark Q24 Vacuum Workstation (Qiagen), then pyrosequencing was done by the PyroMark Q24 system (Qiagen). The global DNA methylation level was quantified with the PyroMark Q24 Software (Qiagen) as the average of the DNA methylation level of 3 CpG sites in LINE-1 sequences. Statistical significances (*p* ≤ 0.05) were assessed by Kruskal–Wallis test followed by Dunn’s multiple comparisons test using Prism8 software (GraphPad).

### 2.7. Whole Genomic DNA Methylation Analysis with Illumina Bead Array Technique

Isolated DNA (~250 ng) was used for methylation status evaluation of over 850,000 methylation sites across the whole genome. The analysis was performed in the Austrian Institute of Technology in Vienna with the assignment of Diagenode Diagnostics (Diagenode, Seraing, Belgium). Briefly, bisulfite conversion was followed by DNA amplification and fragmentation, then DNA was hybridized to the EPIC BeadChip array (Illumina, San Diego, CA, USA). The intensity of methylated and unmethylated beads was detected with scanning after biotin and 2,4-dinitrophenol labeling. Raw files generated by the Illumina pipeline in the IDAT format were processed with the ChAMP (chip analysis methylation pipeline) R package. Initial filtering steps were performed to discard low-quality probes with *p*-value ≥ 0.01 in any of the investigated samples or a bead-count < 3 in at least 5% of all samples. Additionally, non-CpG probes, SNP (single nucleotide polymorphism)-related probes, multi-hit (non-specific) probes and all probes located on chromosomes X or Y were also removed from the dataset. Normalization due to the bias introduced by the inherently different hybridization chemistries of type-I and type-II Illumina probes was performed using the Beta Mixture Quantile dilation technique. Batch effects were investigated, and correction was deemed unnecessary. The remaining 721,878 probes were used for association analyses. Probes for which the methylation level changed in different directions in the two treated groups compared to the untreated samples were deemed ambiguous (non-treatment-related) and discarded. An additional filtering step was also performed to only include probes located on CpG-islands.

Mean methylation levels (β-values) were compared for the remaining probes with t-tests, and Benjamini–Hochberg correction was used to account for multiple testing. Probes with an adjusted *p*-value of 0.05 or lower in any of the above comparisons were deemed to be significantly altered. A probe was defined to be hypo- or hypermethylated if the average methylation level (β-value) in both the 0.5 mmol/L and the 1 mmol/L treatment groups were lower or higher than the average β-value of untreated samples, respectively. Significantly altered probes in both these categories were ordered in a decreasing manner based on the absolute average β-value difference in all treated samples compared to their untreated sample pairs.

### 2.8. ELISA Assay and Immunocytochemistry for γ-H2AX Detection

Quantification of γ-H2AX formation was performed with a High Throughput γ-H2AX ELISA Kit (R&D Systems, Minneapolis, MN, USA) and immunocytochemistry. For ELISA measurement, harvested HT-29 and SW480 cells were washed in 1× PBS and stored at −20 °C. Approximately 500,000 cells/samples were centrifuged, resuspended with Cell Lysis Buffer (R&D Systems), and incubated on ice for 20 min with periodical vortexing. Finally, samples were diluted with Assay Buffer (R&D Systems) and approx. 50,000 cells were transferred to the ELISA plate provided by the manufacturer. Relative light units (RLU) of each well were detected by a Fluoroskan Ascent FL Microplate Fluorometer and Luminometer using Ascent Software (Thermo Fisher Scientific).

For immunocytochemistry, HT-29 and SW480 cells were grown in glass coverslips, placed into 6-well plates as described before by Valcz et al. [27]. Following SAM treatment, cells were fixed with 4% paraformaldehyde for 10 min at 4 °C. Incubation was performed with 2M HCl for 10 min and with 0.2% Triton for another 10 min. The activation of DNA double-strand break repair was detected using an anti-γ-H2AX antibody (Abcam, Cambridge, UK) (1 h; 1:150 dilution; anti-mouse) labeled with Alexa 488 (Invitrogen, Carlsbad, CA, USA) (30 min; 1:200 dilution). Cell nuclei were stained with Hoechst staining (Thermo Fischer Scientific) (5 min; 1:1000 dilution). Coverslips were washed with 1× PBS 3 times for 5 min between each step of the immunostaining. Slides were digitalized with Pannoramic Confocal scanner (3DHISTECH Ltd., Budapest, Hungary) in 21 Z-axial confocal layers of 0.4 μm focus steps. Statistical significances (*p* ≤ 0.05) were assessed by Kruskal–Wallis test followed by Dunn’s multiple comparisons test using Prism8 software (GraphPad).

### 2.9. Genomic Stability Detection with Comet Assay

The SAM-treated cells were removed from the plate with a rubber policeman, then washed with Dulbecco’s Phosphate Buffered Saline (Sigma-Aldrich). A Comet Assay Kit (Abcam) was used according to the manufacturer’s instruction with a minor modification: original slides were replaced with agarose-coated Superfrost Ultra Plus slides (Thermo Fisher Scientific). Electrophoresis was performed at 150 mA for 45 min in alkaline electrophoresis solution. Vista Green Dye was added to each slide to DNA staining. DNA damage was captured by a fluorescence microscope (Carl Zeiss AG, Oberkochen, Germany), equipped with an AxioCam camera (Carl Zeiss AG) and was analyzed using Comet Score software. Tail DNA% was calculated in 50 cells/sample based on the ratio of tail DNA and cell DNA lenght. Statistical significances (*p* ≤ 0.05) were assessed by Kruskal–Wallis test followed by Dunn’s multiple comparisons test using Prism8 software (GraphPad).

### 2.10. Micronuclei Scoring and Staining

An increased number of micronuclei can also indicate the disturbances of mitotic apparatus and/or repair machinery referring to genomic instability. Therefore, immunocytochemistry analysis was applied as described in the above-mentioned paragraph “γ-H2AX detection with ELISA and immunocytochemistry”, and CellQuant tool of the CaseViewer software (3DHISTECH) was used to detect micronucleus number. An area containing approximately 1000 cells per sample was analyzed. Micronuclei were counted and divided by the total cell number to get the percentage of cells owing micronucleus. Statistical significances (set at *p* ≤ 0.05) were assessed by Kruskal–Wallis test followed by Dunn’s multiple comparisons test using Prism8 software (GraphPad).

### 2.11. Quantitative and Qualitative Analysis of Cell-Free DNA with DNA Isolation

Medium of the treated cells (2.5 mL) was collected and used for cell-free DNA (cfDNA) isolation following centrifugation (10 min at 1000 rpm). The cfDNA was extracted with a Quick-cfDNA Serum and Plasma Kit (Zymo Research) according to the manufacturer’s protocol, then sample concentrations were measured with a Qubit dsDNA HS Assay Kit (Thermo Fisher Scientific), and fragment-length analysis was performed with an Agilent High Sensitivity DNA Kit using an Agilent 2100 Bioanalyzer system (Agilent Technologies).

### 2.12. Whole Exome Sequencing

Library preparation was performed by a Nextera DNA Exome kit (Illumina) starting from 50 ng DNA isolated from the treated HT29 and SW480 cells. After tagmentation, indexing and amplification steps according to the manufacturer’s instructions, quantification of the purified libraries was performed with a Qubit 1.0 fluorometer (Thermo Fisher Scientific) using a Qubit dsDNA HS Assay Kit (Thermo Fisher Scientific). Then library fragment size distributions were determined by a Bioanalyzer 2100 instrument (Agilent Technologies) with a High Sensitivity DNA Kit (Agilent Technologies). Next generation sequencing was performed on a NextSeq500 instrument (Illumina) using pools of nine amplified dsDNA libraries with a NextSeq 500/550 High Output Sequencing Kit (150 cycles).

Initial quality assessment of sequencing results was performed with the fastQC and multiQC software. After confirming that the quality metrics of the sequencing were satisfactory, raw FASTQ files were aligned to the hg38 human reference genome sequence with the BWA-MEM tool. Duplicated reads were identified with the Picard MarkDuplicates command and base qualities were adjusted with the BaseRecalibrator and ApplyBQSR tools of the GATK (Genome Analysis Toolkit). Germline mutations were identified in accordance with the GATK “Germline short variant discovery best practice” workflow [28], with the use of the HaplotypeCaller, GenomicsDBImport, GenotypeGVCFs, VariantRecalibrator and ApplyRecalibration tools.

Somatic mutations were detected as outlined in the “Somatic short variant discovery (SNVs + Indels)” best practice of GATK with Mutect2 and FilterMutectCalls tools. A Panel of Normal samples was generated from the whole exome sequencing data of healthy individuals sequenced in the same manner as the investigated samples. All detected mutations were annotated with the vcf2maf and VEP (Ensembl Variant Effect Predictor) software [29]. The dataset of the present study was uploaded to Sequence Read Archive (SRA): SubmissionID: SUB7855933 (https://submit.ncbi.nlm.nih.gov/subs/bioproject/SUB7855933/overview).

## 3. Results

### 3.1. Effect of SAM Treatment on Cell Proliferation and Cell Cycle

**Figure 1:**
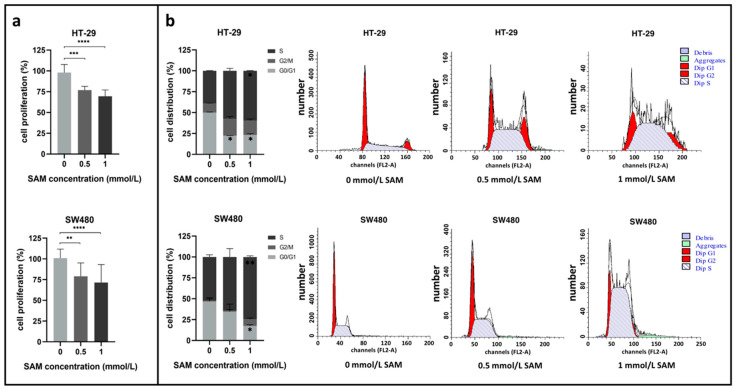
(**a**) Cell proliferation of HT-29 and SW480 cells were analyzed with SRB (sulforhodamine B) assay. The median of control (0 mmol/L S-adenosylmethionine (SAM)) cells was taken as 100% and the average results of the treatment groups (0.5 mmol/L; 1 mmol/L SAM) were compared to this value (** *p* ≤ 0.005, *** *p* ≤ 0.0005, and **** *p* ≤ 0.0001). (**b**) Cell cycle alterations were detected with FACS (fluorescence-activated cell sorting) in HT-29 and SW480 cells. Left: Percentage of cell distribution in different phases of the cell cycle (* *p* ≤ 0.05, ** *p* ≤ 0.005). Right: Representative histograms about each treatment conditions. Dip: diploid.

The impact of SAM treatment on the proliferation of HT-29 and SW480 cell lines was measured with SRB assay. Our results showed that SAM inhibited cell growth significantly (*p* ≤ 0.005) in a concentration-dependent manner in both cell lines (HT-29_ctrl_: 98.08 ± 9.71%, HT-29_0.5_: 76.93 ± 4.48%, HT-29_1_: 69.54 ± 7.63%; SW480_ctrl_: 100.98 ± 10.88%, SW480_0.5_: 79.00 ± 16.11%, SW480_1_: 71.54 ± 21.67%) (Figure 1a).

FACS (fluorescence-activated cell sorting) analysis was performed in order to get a proper insight about the exact time-point of the cell cycle that is mostly involved by SAM. The treatment decreased the proportion of HT-29 and SW480 cells in the G0/G1 phase (HT-29: from 50.00 ± 0.81% to 23.31 ± 1.58%; SW480: from 46.71 ± 4.59% to 17.49 ± 1.49%), while it increased in the S (HT-29: from 39.29 ± 0.28% to 59.49 ± 0.25%; SW480: from 52.12 ± 2.70% to 74.68 ± 1.39%) and G2/M (HT-29: from 10.76 ± 0.53% to 17.21 ± 1.83%; SW480: from 1.16 ± 2.01% to 7.83 ± 1.66%) phases (Figure 1b). The percentage of apoptotic cells remarkably elevated in HT-29 (additional 45.17 ± 12.49%) and slightly elevated in SW480 cell line (additional 8.99 ± 5.82%) following 1 mmol/L SAM treatment (data not shown).

### 3.2. Effect of SAM Treatment on the Expression of Genes Involved in Tumor Progression

SAM is known to have an inhibitory effect on the invasion and metastasis of certain tumors; therefore, expression patterns of genes taking part in these processes were investigated. Firstly, we focused on a gene set compiled by S. Bakhoum et al. [30] from epithelial–mesenchymal transition (EMT)/migration/metastasis genes that were upregulated in the metastatic subpopulation of chromosomally unstable cancer cells.

**Figure 2:**
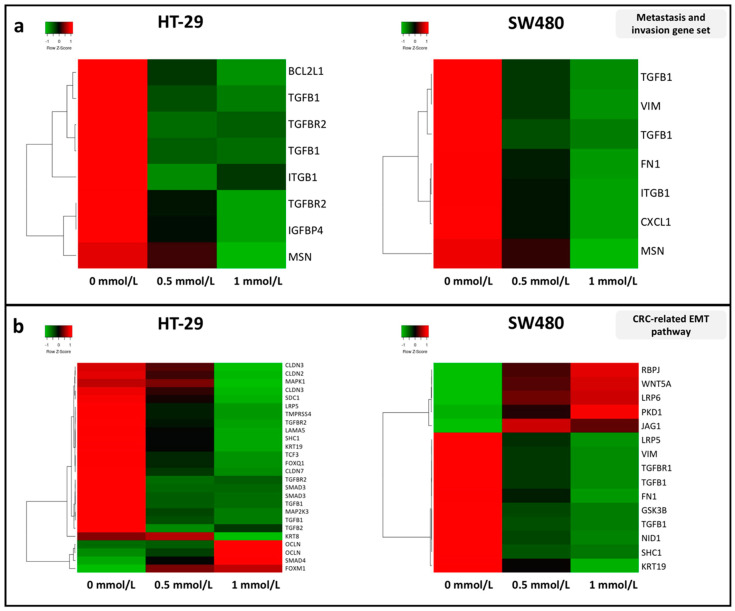
Expression analysis of genes included (**a**) in the gene set compiled by Bakhoum et al. [30] and (**b**) in the epithelial–mesenchymal transition (EMT) pathway comparing control (0 mmol/L) and SAM-treated (0.5 mmol/L; 1 mmol/L) HT-29 (left) and SW480 (right) cells. Transcripts that show significant (*p* ≤ 0.05) alterations with a fold change (FC) ≥ |1.4| in the comparison of 0 and 1 mmol/L SAM treatment were illustrated on heatmaps. Columns are referred to the applied SAM concentration and each row represents a gene transcript probe set on the HTA 2.0 microarray platform (Affymetrix). Green color symbolizes downregulation, while red means increased expression.

All of the transcripts, that met our criteria (fold change (FC) ≥ |1.4|; (*p* ≤ 0.05)) showed decreased level in 1 mmol/L SAM treatment compared to control in both cell lines (Figure 2a). Furthermore, the majority of genes involved in the EMT pathway of colorectal carcinogenesis was examined individually and proved to have downregulated gene expression (Figure 2b).

*TGFB1* is one of the candidates in the most significantly decreasing genes by SAM treatment in both cell lines (1 mmol/L SAM vs. control: FC_HT-29_ = −6.91; FC_SW480_ = −4.52). In HT-29 cells, besides small nucleolar RNA coding genes (*SNORD* and *SNORA*), solely *NTSR1*, *KRT19* and *KLF6*, while in SW480 cells *CCL2* and *ANO1* genes showed larger reduction in their expression than *TGFB1* following SAM treatment (data not shown).

### 3.3. Effect of SAM Treatment on Global and Promoter-Specific DNA Methylation Level

SAM is involved in DNA methylation regulation as a universal methyl donor molecule; therefore, the effect of SAM on global and local DNA methylation level of the two colorectal cancer cell lines with different CIMP statuses was investigated.

**Figure 3:**
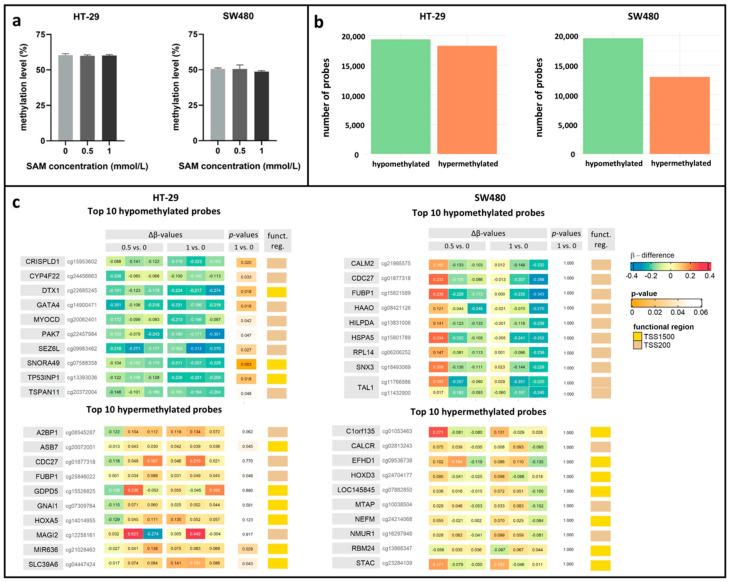
(**a**) Global DNA methylation of control (0 mmol/L) and SAM-treated (0.5 mmol/L; 1 mmol/L) HT-29 (left) and SW480 (right) cells were analyzed by pyrosequencing. DNA methylation status was represented as the average methylation level of three individual CpGs of LINE-1 retrotransposon. (**b**) Promoter-specific methylation of SAM-treated HT-29 (left) and SW480 (right) cells was detected with EPIC BeadChip array. The number of all hypo- and hypermethylated probes located in promoter regions of CpG islands are visualized. (**c**) The top 10 hypo- and hypermethylated probes with the largest absolute mean methylation difference (Δβ) between SAM-treated (0.5 mmol/L; 1 mmol/L) and control (0 mmol/L) HT-29 and SW480 cells were analyzed by EPIC BeadChip array. Funct. reg.: functional region.

Based on the average methylation status of LINE-1 retrotransposons, HT-29 cell line showed almost 10% higher global DNA methylation than SW480 cells. However, after SAM treatment, methylation level of neither HT-29, nor SW480 cells changed significantly (HT-29_ctrl_: 60.40 ± 0.99%, HT-29_0.5_: 59.89 ± 0.68%, HT-29_1_: 60.14 ± 0.64%; SW480_ctrl_: 50.58 ± 0.71%, SW480_0.5_: 52.08 ± 1.32%, SW480_1_: 48.65 ± 0.64%) (Figure 3a).

In the following, we examined DNA methylation patterns of promoter regions localized in CpG islands by the Illumina EPIC BeadChip method in HT-29 and SW480 cell lines. In general, most of the altered cgIDs showed relative hypomethylation after SAM treatment (Figure 3b). We further focused on the top 10 hypo- and hypermethylated cgIDs with the largest absolute mean methylation difference between treated and non-treated cells. The effect of SAM to decrease methylation was proved to be beneficial in certain genes (HT-29: *DTX1* [31], *GATA4* [32], *SEZ6L* [33], *TP53INP1* [34]; SW480: *HAAO* [35], *TAL1* [36]), whose hypermethylation was associated with tumor progression according to the scientific literature (Figure 3c).

### 3.4. Effect of SAM Treatment on Genomic Stability

**Figure 4:**
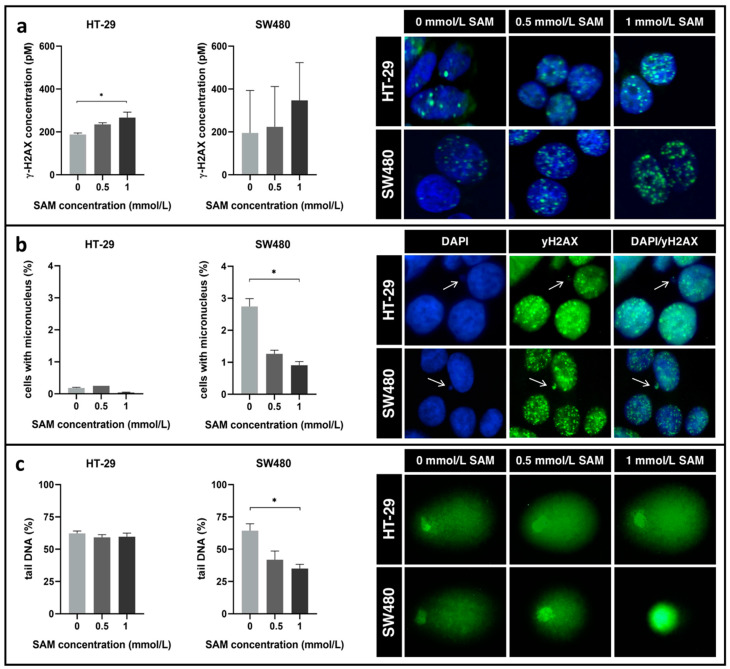
(**a**) Left: Concentration of γ-H2AX was determined with ELISA. Right: Representative immunofluorescence staining images of γ-H2AX intensity following SAM treatment were captured. (**b**) Left: Micronucleus scoring was performed on the slides stained with DAPI and anti-γ-H2AX (* *p* ≤ 0.05). Right: Arrows indicate representative micronuclei with γ-H2AX positivity. (**c**) Left: Percentage of DNA in the tail was referred as comet tail DNA % (* *p* ≤ 0.05). Right: Characteristic comets are visualized following SAM treatment.

Remarkable elevation of γ-H2AX concentration was detected in both cell lines after SAM treatment using ELISA. However, this alteration was not significant in SW480 cells (HT-29_ctrl_: 188.24 ± 7.16 pM, HT-29_0.5_: 235.38 ± 7.42 pM, HT-29_1_: 266.73 ± 25.35 pM; SW480_ctrl_: 195.30 ± 197.94 pM, SW480_0.5_: 223.76 ± 187.85 pM, SW480_1_: 347.30 ± 176.19 pM) (Figure 4a).

To clarify this phenomenon, micronucleus scoring, and comet assays were performed additionally, as decreasing micronucleus number and comet tail shortening refer to DNA repair activation.

Firstly, the genomic stability markers were compared between non-treated SW480 and HT-29 cells. Remarkable differences were noticed in micronuclei formation (HT-29_ctrl_: 0.19%; SW480_ctrl_: 2.75%) (Figure 4b), however, the percentage of comet tail lengths relative to the cell (tail DNA%) fell in the range of 60–70% in the comparison of the two cell lines (Figure 4c).

Although SAM treatment had no considerable effect on HT-29 cells neither in point of micronucleus number (HT-29_ctrl_: 0.19 ± 0.02%, HT-29_0.5_: 0.25 ± 0.00%, HT-29_1_: 0.04 ± 0.01%) (Figure 4b), nor in comet tail length (HT-29_ctrl_: 62.29 ± 1.82%, HT-29_0.5_: 59.2 ± 2.12%, HT-29_1_: 59.69 ± 2.81%) (Figure 4c), it could significantly reduce these parameters in SW480 cells (micronucleus number: SW480_ctrl_: 2.75 ± 0.25%, SW480_0.5_: 1.26 ± 0.12%, SW480_1_: 0.91 ± 0.11% (Figure 4b); comet tail length: SW480_ctrl_: 64.36 ± 5.36%, SW480_0.5_: 41.84 ± 6.75%, SW480_1_: 35.01 ± 3.29%) (Figure 4c) referring to the activation of DNA repair in this particular cell line.

**Figure 5:**
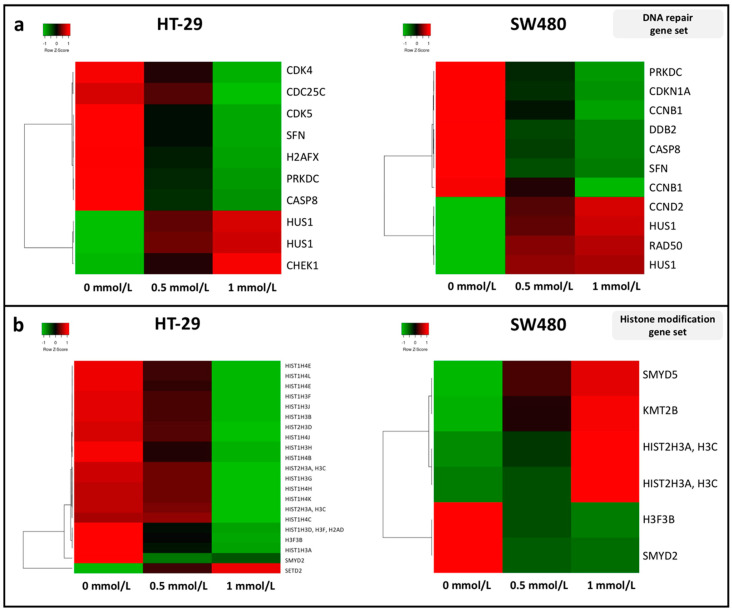
Expression analysis of genes included (**a**) in the DNA damage response pathway and (**b**) in histone modification gene set in control (0 mmol/L) and SAM-treated (0.5 mmol/L; 1 mmol/L) HT-29 (left) and SW480 (right) cells. Transcripts showing significant (*p* ≤ 0.05) alterations with a FC ≥ |1.4| in the comparison of 0 and 1 mmol/L SAM treatment were illustrated on heatmaps. Columns are referred to the applied SAM concentration and each row represents a gene transcript probe-set on the HTA 2.0 microarray platform (Affymetrix). Green color symbolizes downregulation, while red means increased expression.

An expanded list of DNA damage response (DDR) genes was studied by their expression changes following SAM treatment. According to our observations, most of the transcripts were downregulated (e.g., *SFN*, *PRKDC* and *CASP8*) except for *HUS1* in both cell lines (Figure 5a). We analyzed genes involved in histone modification processes, and downregulated gene expression levels (except for *SETD2*) were detected in HT-29 cells compared to SW480, where most of the genes showed elevated expression (Figure 5b). Although in the applied gene sets *H2AFX* was listed as a repair gene, it is important to mention, that this is additionally a histone variant showing decreased expression in HT-29 cells by SAM treatment.

**Figure 6:**
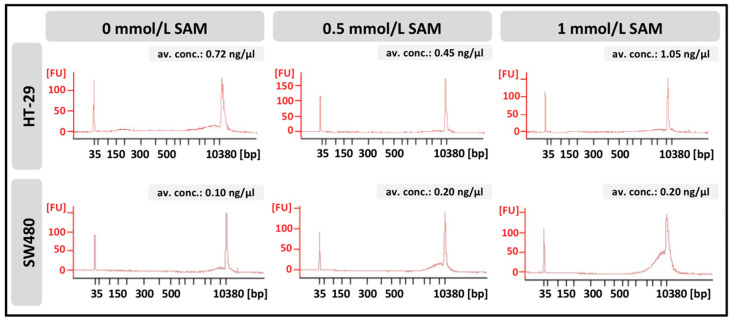
Fragment–length distribution of cell-free DNA isolated from the media of HT-29 and SW480 cells was detected with BioAnalyzer. Electropherograms represent a data plot of size in base pairs (bp) versus fluorescence (FU). Average DNA concentrations were measured with Qubit 1.0 fluorometer. Av. conc.: average concentration.

Analyses of cell-free DNA concentration and fragment–length distribution were performed in the two different cell lines. In general, HT-29 cells showed higher DNA release into the cell culture medium, than SW480 comparing control groups. However, following SAM treatment, no significant accumulation could be observed at any fragment–length ranges in HT-29 cells in contrast to SW480, in which case a remarkable elevation occurred at around 10,000 base pairs (Figure 6).

**Figure 7:**
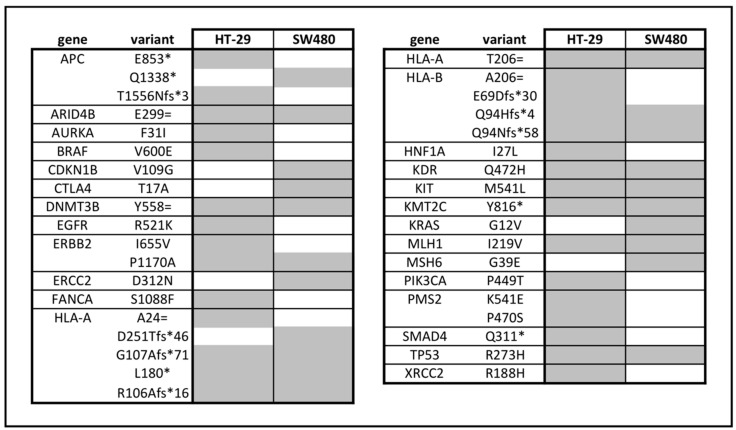
Mutation profile of HT-29 and SW480 cell lines was analyzed by exome sequencing. Intronic variants were excluded, and mutated genes considered as likely oncogenic on the basis of the OnkoKB database were shown. Grey color indicates gene mutation.

To explore the connections between the genetic background of the two non-treated cell lines and their response for SAM treatment, mutations localized in the coding region were analyzed by whole exome sequencing. We focused on likely oncogenic genes annotated by the OnkoKB (www.onkokb.org) database. Many similarities were found in the mutation profile of the two non-treated cell lines (*ARID4B, DNMT3B, ERBB2, HLA-A, HLA-B, KDR, KIT, KMT2C, MLH1, TP53)*, however, only HT-29 was proved to be *BRAF*, *EGFR*, *PIK3CA* mutant, while SW480 had mutations in *KRAS* and *MSH6* genes (Figure 7).

Comparing the two cell lines, HT-29 had 18.42% more mutation count (9979.3 ± 685), than SW480 (8141 ± 53), and new mutations were not observed following SAM treatment (data not shown) in the genes illustrated in Figure 7.

## 4. Discussion

SAM plays a pivotal role in many important biochemical reactions such as transsulfuration, aminopropylation, polyamine synthesis [14,22,37], and the methylation of phospholipids, proteins, RNA and DNA [14,22]. Furthermore, SAM can be useful in the treatment of various diseases (e.g., cholestasis, depression, osteoarthritis) [38,39,40], and it has been proven to inhibit the progression of several tumors [16,17,18,19,20,21]. The latter is primarily attributed to its epigenetic modulatory properties [13,18]; however, recent studies have demonstrated that the involvement of SAM in other metabolic processes is also crucial [19].

The main focus of our study was to examine the effect of 48-hour-long SAM treatment on major pathways (CIN, MSI, CIMP) related to tumorigenesis. In order to observe whether this impact of SAM is dependent on the genetic and epigenetic background of the recipient, we investigated two CRC cell lines with different molecular features. Loss of genomic stability is mainly induced by chromosomal instability in CRC [41], hence we used CIN+ and MSI- cell lines, namely HT-29 and SW480. Besides the similarity of their CIN status, fundamental differences can be identified between the cells, since HT-29 bears *BRAF* and *PIK3CA* mutations, and can be classified into the CIMP+ subgroup, whereas SW480 is a *KRAS* mutant CIMP- cell type. Moreover, HT-29 is sorted into CMS3 group with the characteristics of epithelial and metabolic dysregulation, while SW480 is considered as CMS4, therefore show elevated TGF-β signaling, prominent stromal infiltration and accelerated angiogenesis [42].

SAM has been formerly observed to reduce the proliferation of HT-29 cells and increase apoptotic cell number [13,24]. Our results are in concordance with previous data as both cell lines showed significant decrease (approx. 30%) in proliferation and an approximately 2.5 times elevation in the number of apoptotic cells for 1 mmol/L SAM treatment. Proliferation is critically regulated by the cell cycle machinery, so we took a closer look and observed a SAM-related S phase arrest in both CRC cell lines. This phenomenon is considered to be related to the activation of DNA repair processes [43].

The influence of SAM on gene expression pattern was analyzed in order to investigate the relationship between the attenuation of tumor cell proliferation and the transcriptomic changes. The majority of genes involved in EMT, migration and metastasis showed decreased gene expression after SAM treatment. Among them, *TGFB1* was the most significantly downregulated gene in both cell lines, which is known to promote the progression of advanced neoplasia [44]. Furthermore, clinically relevant genes that are associated with tumor growth such as *VIM* [45], *ITGB1* [46], *FOXQ1* [47], *FN1* [48], *MSN* [49], *CXCL* [50] and *TMPRSS4* [51] were also downregulated. Thereby, these findings suggest that SAM can be efficient in the inhibition of tumor spreading in gene expression level. Supposedly, further *in vivo* studies would provide interesting information about how SAM treatment could alter the metastatic potential of these cell lines.

The observed gene expression alterations might be due to the silencing effect of epigenetic modifications [13]. Consequently, this phenomenon has inspired us to examine the impact of SAM treatment on the global DNA methylation pattern in the two CRC cell lines. The comparison of the non-treated control groups revealed that HT-29 cells had higher methylation level than SW480 cells in agreement with their different CIMP status. However, according to our experiments, SAM treatment did not result in a significant global methylation change in any of the cell lines. These data seem to be consistent with the hypothesis suggested by Wang et al., that SAM might rather consider to be a methylome modulator, then a pure hypermethylating agent [52]. CpG-site specific methylation changes of promoter regions (TSS1500, TSS200) were also examined after SAM treatment, and interestingly the majority of the investigated probes showed hypomethylation in both cell lines. We assume that three main factors may underlie this observation. Firstly, SAM is known to be a methyl donor molecule, but its involvement in various biochemical reactions may counteract with the DNA methylating effect. For instance, SAM is converted into S-adenosyl-L-homocysteine (SAH), which inhibits DNA methyltransferase enzymes, thus reducing DNA methylation [53]. Secondly, in our study, we were treating cancer cells, and due to their defective metabolism, SAM might be used differently compared to normal cells. Lastly, we suppose that the short-term (48-hour-long) SAM treatment may have another effect as do long-term ones, so *in vivo* experiments would be necessary to clarify this phenomenon.

The decreased methylation of the investigated TSS1500 and TSS200 regions might be beneficial in some cases, as hypermethylation were in concordance with tumor progression in certain genes (*DTX1* [31], *GATA4* [32], *SEZ6L* [33], *TP53INP1* [34], *HAAO* [35], *TAL1* [36]) associated with the top 10 hypomethylated probes localized in promoters. However, promoter methylation of the top 10 hypermethylated genes has not been reported to take part in the inhibition of tumor progression yet. Despite the fact that elevated DNA methylation level in the promoter regions is often accompanied by decreased gene expression [13], according to our results, none of the examined genes showed lower gene expression. In parallel, based on our study, reduced expression of genes involved in tumor growth might not be directly influenced by DNA methylation level changes. Thereby, it is likely that SAM does not exert its primary effect on the analyzed cancer cell lines via DNA methylation. This assumption has also been concluded by Mahmood et al. [54] and Schmidt at al. [19] with the application of SAM on breast and prostate cancer models, respectively.

As Sheaffer et al. described, tumor progression is accompanied with decreased global DNA methylation level leading to genomic instability [55]. SAM can reverse the hypomethylation of certain genes [56], thereby presumably affecting genomic stability as well. Finding S phase arrest in both cell lines by SAM treatment, activation of repair processes was suspected and investigated with several methods. Double-stranded DNA breaks induce H2AX histone phosphorylation, thereby the formation of γ-H2AX in order to facilitate the recruitment of repair proteins to the damaged site [57]. According to our results, characteristic elevation of γ-H2AX concentration was observed in both cell lines after SAM treatment, that was only significant in HT-29 cells. This could account for either the increased number of DNA breaks or the activation of repair mechanisms [57]. To determine the accurate mechanism, micronucleus and comet assays were accomplished. Micronuclei formation is a consequence of chromosomal breaks or the disruption of the mitotic apparatus [58]. SAM has been previously described by Ramírez et al. to play a major role in chromatin segregation and condensation, supposedly via the restoration of microtubule methylation. Therefore, it is able to reduce the frequency of micronuclei formation originated from chromosome loss during cell division [59,60]. Based on our study, significant reduction in micronuclei number of SW480 cells has been detected by 1 mmol/L SAM treatment. Comets can be visualized by the elevated migration of DNA segments originated from broken strands [61]. In SW480 cells, just like in the case of micronuclei scoring, a significant decrease in comet tail length was detected by 1 mmol/L SAM treatment. Therefore, our findings suggest that SAM-induced repair processes in this cell line were activated. Interestingly, at the point of genomic stability no remarkable alterations were found in HT-29 by SAM treatment. 

Comparing non-treated cells, comet tail lengths proved to be in the same range; however, the micronucleus number was lower in HT-29 cells, than in SW480. Micronucleus count is inversely correlated with DNA methylation and influenced by the proliferation rate as well [61,62]. Therefore, the difference between the two cell lines might be explained by the fact that SW480 has a lower DNA methylation level, and it is a more actively proliferating cell type than HT-29.

In the background of the variously inducible DNA repair mechanisms between the two cell lines, we hypothesized that alterations in the expression of DDR genes occurred. The majority of the transcripts were downregulated except for the overexpression of *CHEK1* in HT-29 *CCND2* along with the *RAD50* in SW480 and *HUS1* gene in both cell lines. The SAM pathway is involved in DNA folding, which is important for proper DNA repair [63]. Therefore, concerning genomic stability, it is necessary to study the expression of either genes that code histones or proteins that modify them. Even though the majority of histone-coding genes, including *H2AFX*, are expressed primarily during the S phase [64] (where more than half of the cells were accumulated), we observed a significant downregulation of these transcripts in HT-29 after 1 mmol/L SAM treatment. However, in SW480 cells the majority of significantly altered genes in the “histone modification” pathway were upregulated, which potentially indicates the proper formation of chromosomes during proliferation. Interestingly, in SW480 cells accumulation of cell-free DNA fragments with a length of 10,000 base pairs appeared after SAM treatment in contrast to HT-29 cells, where this phenomenon could not be observed. It was reported that micronuclei can be released from the cells, which may lead to chromosome loss [65]. Therefore, we suppose that the cell-free DNA might be originated from micronuclei. The oncogenic mutation profile of the two CRC cell lines was also examined in order to investigate its role in the maintenance of genomic stability. Opposing to SW480, HT-29 cells proved to have mutations in *FANCA* [66], *PMS2* [67], *XRCC2* [68] DDR genes, that may also contribute to the differences in their DNA repair capabilities.

## 5. Conclusions

Overall, we can conclude that SAM may inhibit the progression of HT-29 and SW480 cells by modulating gene expression. This effect is not closely related to its ability to remethylate DNA; therefore, we suppose that the role of SAM in many other biochemical reactions is responsible for this phenomenon. Our study provides evidence for the first time, that SAM can also induce the activation of DNA repair processes in SW480 cells.

SAM is required for normal cell function and has limited documented toxicities; therefore, it has long been favorably used as a dietary supplement. Our results indicate that its use in tumor therapy as adjunctive medication to chemotherapeutical agents is definitely worth considering. However, the application of molecular diagnostics before SAM treatment is important as our experiment has also highlighted, it may have different effects on tumors depending on their genetic and epigenetic properties.

## References

[1] Bray F., Ferlay J., Soerjomataram I., Siegel R.L., Torre L.A., Jemal A. (2018). Global cancer statistics 2018: GLOBOCAN estimates of incidence and mortality worldwide for 36 cancers in 185 countries. CA Cancer J. Clin..

[2] Yao Y., Dai W. (2014). Genomic Instability and Cancer. J. Carcinog Mutagen.

[3] Shen Z. (2011). Genomic instability and cancer: An introduction. J. Mol. Cell Biol..

[4] Włodarczyk M., Włodarczyk J., Siwiński P., Sobolewska-Włodarczyk A., Fichna J. (2018). Genetic Molecular Subtypes in Optimizing Personalized Therapy for Metastatic Colorectal Cancer. Curr. Drug Targets.

[5] Smeby J., Sveen A., Merok M.A., Danielsen S.A., Eilertsen I.A., Guren M.G., Dienstmann R., Nesbakken A., Lothe R.A. (2018). CMS-dependent prognostic impact of KRAS and BRAFV600E mutations in primary colorectal cancer. Ann. Oncol..

[6] Grady W.M. (2004). Genomic instability and colon cancer. Cancer Metastasis Rev..

[7] Markowitz S.D., Bertagnolli M.M. (2009). Molecular Basis of Colorectal Cancer. N. Engl. J. Med.

[8] Guinney J., Dienstmann R., Wang X., de Reyniès A., Schlicker A., Soneson C., Marisa L., Roepman P., Nyamundanda G., Angelino P. (2015). The consensus molecular subtypes of colorectal cancer. Nat. Med..

[9] Kulis M., Esteller M. (2010). DNA methylation and cancer. Adv Genet..

[10] Meaney M.J., Szyf M. (2005). Environmental programming of stress responses through DNA methylation: Life at the interface between a dynamic environment and a fixed genome. Dialogues Clin. Neurosci..

[11] Das P.M., Singal R. (2004). DNA methylation and cancer. J. Clin. Oncol..

[12] Gautrey H.E., van Otterdijk S.D., Cordell H.J., Mathers J.C., Strathdee G. (2014). DNA methylation abnormalities at gene promoters are extensive and variable in the elderly and phenocopy cancer cells. FASEB J..

[13] Luo J., Li Y.N., Wang F., Zhang W.M., Geng X. (2010). S-adenosylmethionine inhibits the growth of cancer cells by reversing the hypomethylation status of c-myc and H-ras in human gastric cancer and colon cancer. Int. J. Biol. Sci..

[14] Lu S.C. (2000). S-Adenosylmethionine. Int. J. Biochem. Cell Biol..

[15] Gören J.L., Stoll A.L., Damico K.E., Sarmiento I.A., Cohen B.M. (2004). Bioavailability and lack of toxicity of S-adenosyl-L-methionine (SAMe) in humans. Pharmacotherapy.

[16] Parashar S., Cheishvili D., Arakelian A., Hussain Z., Tanvir I., Khan H.A., Szyf M., Rabbani S.A. (2015). S-adenosylmethionine blocks osteosarcoma cells proliferation and invasion in vitro and tumor metastasis in vivo: Therapeutic and diagnostic clinical applications. Cancer Med..

[17] Stoyanov E., Mizrahi L., Olam D., Schnitzer-Perlman T., Galun E., Goldenberg D.S. (2017). Tumor-suppressive effect of S-adenosylmethionine supplementation in a murine model of inflammation-mediated hepatocarcinogenesis is dependent on treatment longevity. Oncotarget.

[18] Zhao Y., Li J.S., Guo M.Z., Feng B.S., Zhang J.P. (2010). Inhibitory effect of S-adenosylmethionine on the growth of human gastric cancer cells in vivo and in vitro. Chin. J. Cancer.

[19] Schmidt T., Leha A., Salinas-Riester G. (2016). Treatment of prostate cancer cells with S-adenosylmethionine leads to genome-wide alterations in transcription profiles. Gene.

[20] Pakneshan P., Szyf M., Farias-Eisner R., Rabbani S.A., Rabbani S.A. (2004). Reversal of the hypomethylation status of urokinase (uPA) promoter blocks breast cancer growth and metastasis. J. Biol. Chem..

[21] Guruswamy S., Swamy M.V., Choi C.I., Steele V.E., Rao C.V. (2008). S-adenosyl L-methionine inhibits azoxymethane-induced colonic aberrant crypt foci in F344 rats and suppresses human colon cancer Caco-2 cell growth in 3D culture. Int. J. Cancer.

[22] Loenen W.A.M. (2006). S-Adenosylmethionine: Jack of all trades and master of everything?. Biochem. Soc. Trans..

[23] Berg K.C.G., Eide P.W., Eilertsen I.A., Johannessen B., Bruun J., Danielsen S.A., Bjørnslett M., Meza-Zepeda L.A., Eknæs M., Lind G.E. (2017). Multi-omics of 34 colorectal cancer cell lines—a resource for biomedical studies. Mol. Cancer.

[24] Li T.W.H., Zhang Q., Oh P., Xia M., Chen H., Bemanian S., Lastra N., Circ M., Moyer M.P., Mato J.M. (2009). S-Adenosylmethionine and methylthioadenosine inhibit cellular FLICE inhibitory protein expression and induce apoptosis in colon cancer cells. Mol. Pharmacol..

[25] Yang H., Sadda M.R., Li M., Zeng Y., Chen L., Bae W., Ou X., Runnegar M.T., Mato J.M., Lu S.C. (2004). S-adenosylmethionine and its metabolite induce apoptosis in HepG2 cells: Role of protein phosphatase 1 and Bcl-x(S). Hepatology.

[26] Kalmár A., Nagy Z.B., Galamb O., Csabai I., Bodor A., Wichmann B., Valcz G., Barták B.K., Tulassay Z., Igaz P. (2019). Genome-wide expression profiling in colorectal cancer focusing on lncRNAs in the adenoma-carcinoma transition. BMC Cancer.

[27] Valcz G., Buzás E.I., Kittel Á., Krenács T., Visnovitz T., Spisák S., Török G., Homolya L., Zsigrai S., Kiszler G. (2019). En bloc release of MVB-like small extracellular vesicle clusters by colorectal carcinoma cells. J. Extracell Vesicles.

[28] Van der Auwera G.A., Carneiro M.O., Hartl C., Poplin R., Del Angel G., Levy-Moonshine A., Jordan T., Shakir K., Roazen D., Thibault J. (2013). From FastQ data to high confidence variant calls: The Genome Analysis Toolkit best practices pipeline. Curr. Protoc. Bioinform..

[29] McLaren W., Gil L., Hunt S.E., Riat H.S., Ritchie G.R., Thormann A., Flicek P., Cunningham F. (2016). The Ensembl Variant Effect Predictor. Genome Biol..

[30] Bakhoum S.F., Ngo B., Laughney A.M., Cavallo J.A., Murphy C.J., Ly P., Shah P., Sriram R.K., Watkins T.B.K., Taunk N.K. (2018). Chromosomal instability drives metastasis through a cytosolic DNA response. Nature.

[31] Gaykalova D.A., Zizkova V., Guo T., Tiscareno I., Wei Y., Vatapalli R., Hennessey P.T., Ahn J., Danilova L., Khan Z. (2017). Integrative computational analysis of transcriptional and epigenetic alterations implicates DTX1 as a putative tumor suppressor gene in HNSCC. Oncotarget.

[32] Tao Y.F., Fang F., Hu S.Y., Lu J., Cao L., Zhao W.L., Xiao P.F., Li Z.H., Wang N.N., Xu L.X. (2015). Hypermethylation of the GATA binding protein 4 (GATA4) promoter in Chinese pediatric acute myeloid leukemia. BMC Cancer.

[33] Sepulveda J.L., Gutierrez-Pajares J.L., Luna A., Yao Y., Tobias J.W., Thomas S., Woo Y., Giorgi F., Komissarova E.V., Califano A. (2016). High-definition CpG methylation of novel genes in gastric carcinogenesis identified by next-generation sequencing. Mod. Pathol..

[34] Weng W., Yang Q., Huang M., Qiao Y., Xie Y., Yu Y., jing A., Li Z. (2011). c-Myc inhibits TP53INP1 expression via promoter methylation in esophageal carcinoma. Biochem. Biophys. Res. Commun..

[35] Huang Y.W., Luo J., Weng Y.I., Mutch D.G., Goodfellow P.J., Miller D.S., Huang T.H.M. (2010). Promoter hypermethylation of CIDEA, HAAO and RXFP3 associated with microsatellite instability in endometrial carcinomas. Gynecol. Oncol..

[36] Musialik E., Bujko M., Kober P., Wypych A., Gawle-Krawczyk K., Matysiak M., Siedlecki J.A. (2015). Promoter methylation and expression levels of selected hematopoietic genes in pediatric B-cell acute lymphoblastic leukemia. Blood Res..

[37] Mato J.M., Corrales F.J., Lu S.C., Avila M.A. (2002). S-Adenosylmethionine: A control switch that regulates liver function. FASEB J..

[38] Li N., Zhang H.H., Wang S.H., Zhu W.M., Ren J.A., Li J.S. (2002). S-adenosylmethionine in treatment of cholestasis after total parenteral nutrition: Laboratory investigation and clinical application. Hepatobiliary Pancreat Dis. Int..

[39] Silveri M.M., Parow A.M., Villafuerte R.A., Damico K.E., Goren J., Stoll A.L., Cohen B.M., Renshaw P.F. (2003). S-adenosyl-L-methionine: Effects on brain bioenergetic status and transverse relaxation time in healthy subjects. Biol. Psychiatry.

[40] Soeken K.L., Lee W.L., Bausell R.B., Agelli M., Berman B.M. (2002). Safety and efficacy of S-adenosylmethionine (SAMe) for osteoarthritis. J. Fam. Pract..

[41] Pino M.S., Chung D.C. (2010). The chromosomal instability pathway in colon cancer. Gastroenterology.

[42] Menter D.G., Davis J.S., Broom B.M., Overman M.J., Morris J., Kopetz S. (2019). Back to the Colorectal Cancer Consensus Molecular Subtype Future. Curr. Gastroenterol. Rep..

[43] Iyer D.R., Rhind N. (2017). The Intra-S Checkpoint Responses to DNA Damage. Genes.

[44] Principe D.R., Doll J.A., Bauer J., Jung B., Munshi H.G., Bartholin L., Pasche B., Lee C., Grippo P.J. (2014). TGF-β: Duality of function between tumor prevention and carcinogenesis. J. Natl. Cancer I.

[45] Du L., Li J., Lei L., He H., Chen E., Dong J., Yang J. (2018). High Vimentin Expression Predicts a Poor Prognosis and Progression in Colorectal Cancer: A Study with Meta-Analysis and TCGA Database. Biomed. Res. Int..

[46] Liu Q.Z., Gao X.H., Chang W.J., Gong H.F., Fu C.G., Zhang W., Cao G.W. (2015). Expression of ITGB1 predicts prognosis in colorectal cancer: A large prospective study based on tissue microarray. Int. J. Clin. Exp. Pathol..

[47] Vu T., Datta P.K. (2017). Regulation of EMT in Colorectal Cancer: A Culprit in Metastasis. Cancers.

[48] Cai X., Liu C., Zhang T.N., Zhu Y.W., Dong X., Xue P. (2018). Down-regulation of FN1 inhibits colorectal carcinogenesis by suppressing proliferation, migration, and invasion. J. Cell Biochem..

[49] Valcz G., Galamb O., Krenács T., Spisák S., Kalmár A., Patai Á.V., Wichmann B., Dede K., Tulassay Z., Molnár B. (2016). Exosomes in colorectal carcinoma formation: ALIX under the magnifying glass. Mod. Pathol..

[50] Zhuo C., Wu X., Li J., Hu D., Jian J., Chen C., Zheng X., Yang C. (2018). Chemokine (C-X-C motif) ligand 1 is associated with tumor progression and poor prognosis in patients with colorectal cancer. Biosci. Rep..

[51] de Aberasturi A.L., Calvo A. (2015). TMPRSS4: An emerging potential therapeutic target in cancer. Br. J. Cancer.

[52] Wang Y., Sun Z., Szyf M. (2017). S-adenosyl-methionine (SAM) alters the transcriptome and methylome and specifically blocks growth and invasiveness of liver cancer cells. Oncotarget.

[53] Isakovic L., Saavedra O.M., Llewellyn D.B., Claridge S., Zhan L., Bernstein N., Vaisburg A., Elowe N., Petschner A.J., Rahil J. (2009). Constrained (l-)-S-adenosyl-l-homocysteine (SAH) analogues as DNA methyltransferase inhibitors. Bioorg. Med. Chem. Lett..

[54] Mahmood N., Cheishvili D., Arakelian A., Tanvir I., Khan H.A., Pépin A.S., Szyf M., Rabbani S.A. (2017). Methyl donor S-adenosylmethionine (SAM) supplementation attenuates breast cancer growth, invasion, and metastasis in vivo; therapeutic and chemopreventive applications. Oncotarget.

[55] Sheaffer K.L., Elliott E.N., Kaestner K.H. (2016). DNA Hypomethylation Contributes to Genomic Instability and Intestinal Cancer Initiation. Cancer Prev. Res..

[56] Detich N., Hamm S., Just G., Knox J.D., Szyf M. (2003). The methyl donor S-Adenosylmethionine inhibits active demethylation of DNA: A candidate novel mechanism for the pharmacological effects of S-Adenosylmethionine. J. Biol. Chem..

[57] Kuo L.J., Yang L.X. (2008). Gamma-H2AX—A novel biomarker for DNA double-strand breaks. In Vivo.

[58] Samanta S., Dey P. (2012). Micronucleus and its applications. Diagn Cytopathol..

[59] Ramírez T., García-Montalvo V., Wise C., Cea-Olivares R., Poirier L.A., Herrera L.A. (2003). S-adenosyl-L-methionine is able to reverse micronucleus formation induced by sodium arsenite and other cytoskeleton disrupting agents in cultured human cells. Mutat Res..

[60] Ramírez T., Stopper H., Hock R., Herrera L.A. (2007). Prevention of aneuploidy by S-adenosyl-methionine in human cells treated with sodium arsenite. Mutat Res..

[61] Araldi R.P., de Melo T.C., Mendes T.B., de Sá Júnior P.L., Nozima B.H., Ito E.T., de Carvalho R.F., de Souza E.B., de Cassia Stocco R. (2015). Using the comet and micronucleus assays for genotoxicity studies: A review. Biomed. Pharm..

[62] Luzhna L., Kathiria P., Kovalchuk O. (2013). Micronuclei in genotoxicity assessment: From genetics to epigenetics and beyond. Front. Genet..

[63] Turgeon M.O., Perry N.J.S., Poulogiannis G. (2018). DNA Damage, Repair, and Cancer Metabolism. Front. Oncol..

[64] Li X., Egervari G., Wang Y., Berger S.L., Lu Z. (2018). Regulation of chromatin and gene expression by metabolic enzymes and metabolites. Nat. Rev. Mol. Cell Biol..

[65] Terradas M., Martín M., Tusell L., Genescà A. (2010). Genetic activities in micronuclei: Is the DNA entrapped in micronuclei lost for the cell?. Mutat Res..

[66] Benitez A., Liu W., Palovcak A., Wang G., Moon J., An K., Kim A., Zheng K., Zhang Y., Bai F. (2018). FANCA Promotes DNA Double-Strand Break Repair by Catalyzing Single-Strand Annealing and Strand Exchange. Mol. Cell.

[67] Hendriks Y.M., Jagmohan-Changur S., van der Klift H.M., Morreau H., van Puijenbroek M., Tops C., van Os T., Wagner A., Ausems M.G.F.M., Gomez E. (2006). Heterozygous mutations in PMS2 cause hereditary nonpolyposis colorectal carcinoma (Lynch syndrome). Gastroenterology.

[68] Johnson R.D., Liu N. (1999). Fau-Jasin, M.; Jasin, M. Mammalian XRCC2 promotes the repair of DNA double-strand breaks by homologous recombination. Nature.

